# Atmospheric Pressure Plasma-Mediated Synthesis of Platinum Nanoparticles Stabilized by Poly(vinylpyrrolidone) with Application in Heat Management Systems for Internal Combustion Chambers

**DOI:** 10.3390/nano8080619

**Published:** 2018-08-15

**Authors:** Anna Dzimitrowicz, Piotr Cyganowski, Pawel Pohl, Dorota Jermakowicz-Bartkowiak, Dominik Terefinko, Piotr Jamroz

**Affiliations:** 1 Department of Analytical Chemistry and Chemical Metallurgy, Faculty of Chemistry, Wroclaw University of Science and Technology, Wybrzeze St. Wyspianskiego 27, 50-370 Wroclaw, Poland; pawel.pohl@pwr.edu.pl (P.P.); terefinko.dominik@gmail.com (D.T.); piotr.jamroz@pwr.edu.pl (P.J.); 2 Department of Polymer and Carbonaceous Materials, Faculty of Chemistry, Wroclaw University of Science and Technology, Wybrzeze St. Wyspianskiego 27, 50-370 Wroclaw, Poland; piotr.cyganowski@pwr.edu.pl (P.C.); dorota.jermakowicz-bartkowiak@pwr.edu.pl (D.J.-B.)

**Keywords:** direct current atmospheric pressure glow discharge, heat transfer, nanostructures, plasma–liquid interactions, stabilizer

## Abstract

Poly(vinylpyrrolidone)-stabilized Pt nanoparticles (PVP-PtNPs) were produced in a continuous-flow reaction-discharge system by application of direct current atmospheric pressure glow discharge (dc-APGD) operated between the surface of a flowing liquid anode (FLA) and a pin-type tungsten cathode. Synthesized PVP-PtNPs exhibited absorption across the entire UV/Vis region. The morphology and elemental composition of PVP-PtNPs were determined with transmission electron microscopy (TEM) and energy dispersive X-ray scattering (EDX), respectively. As assessed by TEM, PVP-PtNPs were approximately spherical in shape, with an average size of 2.9 ± 0.6 nm. EDX proved the presence of Pt, C, and O. Dynamic light scattering (DLS) and attenuated total reflectance Fourier transform-infrared spectroscopy (ATR FT-IR) confirmed PtNPs functionalization with PVP. As determined by DLS, the average size of PtNPs stabilized by PVP was 111.4 ± 22.6 nm. A fluid containing resultant PVP-PtNPs was used as a heat conductive layer for a spiral radiator managing heat generated by a simulated internal combustion chamber. As compared to water, the use of PVP-PtNPs enhanced efficiency of the system, increasing the rate of heat transfer by 80% and 30% during heating and cooling, respectively.

## 1. Introduction

Plasma is one of the four states of matter [1]. Due to the unique properties of atmospheric pressure plasmas (APPs), for example, their highly non-equilibrium state, this type of plasma has been used in many fields of science. These applications include, for example, treatment of skin diseases or tissue engineering in biomedicine [2,3,4], detection of elements in analytical chemistry [5], activation of seeds to stimulate plant growth in agriculture [6], decomposition of diluted organic compounds in air in environmental protection [7], and fabrication of nanomaterials in nanotechnology and material engineering [1,8,9]. One area of application in material engineering is synthesis of inorganic nanoparticles (NPs), such as platinum nanoparticles (PtNPs), which display special physicochemical, thermal, and catalytic properties [10]. PtNPs are widely utilized as catalysts in numerous chemical reactions, such as conversion of hydrocarbons [11] and oxygen reduction reactions [12].

Application of APP-mediated methods for production of PtNPs has numerous advantages. These methods rely on generation of reactive oxygen and nitrogen species (RONS), solvated electrons, as well as UV radiation and heat. All these factors facilitate formation of NPs through reduction of metallic precursor compounds, eliminating the need for additional reducing agents, such as hydrazine [13] or sodium borohydrate [14]. To the best of our knowledge, only a few research groups have attempted to synthesize PtNPs by APP [15,16,17,18,19,20]. Koo et al. [15] and Shim et al. [16] produced Pt nanostructures using atmospheric pressure alternating current (ac) H_2_/He discharge generated in contact with a solution of H_2_PtCl_6_. Synthesized PtNPs exhibited an average size of 2 nm and 3–5 nm, respectively [15,16]. Hu et al. [17] synthesized carbon-supported PtNPs by plasma sputtering in water. Similar to PtNPs obtained by Koo et al. [15], PtNPs produced by Hu et al. [17] were 2 nm in size. Comparable results were obtained by Sato et al. [18], who produced PtNPs of 3–10 nm in size by applying microwave-induced plasma generated in liquid. Ichin et al. [19] reported deposition of PtNPs on carbon nanoballs in an aqueous solution of H_2_PtCl_6_ using poly(vinylpyrrolidone) (PVP) or sodium dodecyl sulfate (SDS) as protection agents. The size of PtNPs produced with the aid of PVP and SDS were, on average, 17.5 and 23.0 nm, respectively. Finally, Dao et al. [20] applied radio frequency (rf) atmospheric pressure discharge operated in Ar for production of nanomaterials for flexible dye-sensitized solar cells. Designed nanomaterials consisted of 2–3 nm PtNPs synthesized by a two-step protocol. In the first step, the PtNPs precursor was partially reduced by alcoholic reduction to Pt atoms, following which the reduction process was completed with the aid of APP [20]. 

The main limitation of all abovementioned reaction-discharge systems used for production of PtNPs is that they worked in a non-continuous-flow mode. To increase the rate of production of metallic nanostructures, our group has developed continuous-flow reaction-discharge systems. These systems relied on either direct current atmospheric pressure glow discharge (dc-APGD) or pulse-modulated radio frequency atmospheric pressure glow discharge (pm-rf-APGD) as APP sources. Both types of APP were operated between the surface of solutions of flowing liquid electrodes and pin-type tungsten electrodes [8,9,21,22,23].

A necessary consideration in synthesis of NPs is to ensure that they are highly stable in the dispersing medium. Aggregation, agglomeration, and coalescence are inconvenient processes that commonly result from short interparticle distances between NPs, leading them to attract each other through van der Waals interactions [24]. To prevent these phenomena, repulsive forces in the colloidal phase are necessary to counter van der Waals interactions and increase the stability of isolated NPs [24]. This can be accomplished through selection of a proper capping agent.

Successfully stabilized PtNPs can be used as nanofluids (NFs) for improved transfer of energy in heat management systems (HMSs) [25,26]. HMSs are applied in internal combustion engines, where combustion chambers must operate at extremely high temperatures, reaching 2500 K [27], which result in the oxidation and degradation of the materials used in engines [28]. HMSs can help protect engines by dissipating all waste heat, or by recovering it by transformation into yet another form of energy.

The most popular HMS, used for cooling internal combustion chambers, involves application of a cooling liquid that circulates between header tanks and a radiator [29]. Performance of this type of HMS is achieved by passing the cooling liquid through the combustion chamber block and a set of narrow channels, where it is air-cooled. To increase efficiency of this system, the surface of heat exchange has to be increased; however, this approach is limited because the radiator has to be compact. An alternative route is to apply water-based cooling liquids of improved heat transfer, including NFs [30,31]. For instance, Al_2_O_3_ or CuNPs might facilitate cooling of the internal combustion block by increasing waste heat transfer from 45% to 80%, as compared to water [29,32]. This increased heat transfer efficiency results in increased combustion efficiency and decreased fuel consumption. However, the volume of NF-based cooling liquid required to manage heat of internal combustion engines often exceeds 5 L, while the volume needed to manage heat of internal combustion chambers in power plants can be counted in tens of thousands of liters. This may raise serious problems related to the large scale of such systems and their proper sealing that can prevent potential emission of NPs to the environment. For this reason, a suitable HMS should be characterized by great efficiency and a limited concentration of NPs present in cooling liquid. The answer to these problems may be application of NFs containing NPs of noble metals, because they are extremely effective heat conductors even at ultra-low concentrations, i.e., 0.001% [26,33]. In our previous work, it was demonstrated that polymer-supported AuNPs facilitated heat transfer at a 300% higher rate than water [8]. It could be believed that application of PtNPs could also provide enhanced heat transfer, while their stabilization with PVP would make suitable NFs for cooling combustion chambers. Since the price of Pt is relatively high, PtNPs-based NFs seems to not be suitable for application as cooling liquids on a large scale. Therefore, a HMS is proposed in the present study, where the NF is used not as a mobile phase but as a heat conductive layer, immobilized and sealed within a radiator.

The main objective of this work was to develop a fast and effective plasma-mediated method for synthesis of stable-in-time and monodisperse PVP-coated PtNPs on the basis of dc-APGD, generated between the surface of a flowing liquid anode (FLA) solution and a pin-type tungsten cathode, and operated in a continuous-flow reaction-discharge system. Synthesized PtNPs were characterized by UV/Vis absorption spectrophotometry (UV/Vis) and transmission electron microscopy (TEM) supported by energy-dispersive X-ray scattering (EDX). To confirm surface functionalization of resultant PtNPs by PVP, dynamic light scattering (DLS) and attenuated total reflectance Fourier transform-infrared spectroscopy (ATR FT-IR) were used. PVP-PtNPs were then applied in the form of a NF in a HMS of simulated internal combustion chambers. The NF was used as a conductive layer for dissipating heat from a circulating liquid used for two scenarios: (i) managing heat of the simulated combustion chamber, and (ii) emergency cooling thereof. To the best of our knowledge, this is the first work in which PVP-PtNPs were produced by dc-APGD generated in a continuous-flow reaction discharge-system and then applied in the HMS for internal combustion chambers.

## 2. Materials and Methods

### 2.1. Reagents and Solutions

Chloroplatinic acid hydrate (H_2_PtCl_6_·H_2_O, Sigma-Aldrich, Steinheim, Germany) was used to prepare a stock solution of 1000 mg L^−1^ of Pt(IV) ions. A working solution of 50 mg L^−1^ of Pt(IV) ions was prepared by appropriately diluting the stock solution. Next, 0.25 g of solid poly(vinylpyrrolidone) (PVP, MW = 40,000, Sigma-Aldrich, Steinheim, Germany) was mixed with 1000 mL of the working solution of Pt(IV) ions, giving a final PVP concentration of 0.25% (*m*/*v*). All reagents were of analytical grade or better. Re-distilled water was used throughout.

### 2.2. One-Step Synthesis of PVP-PtNPs

**Figure 1:**
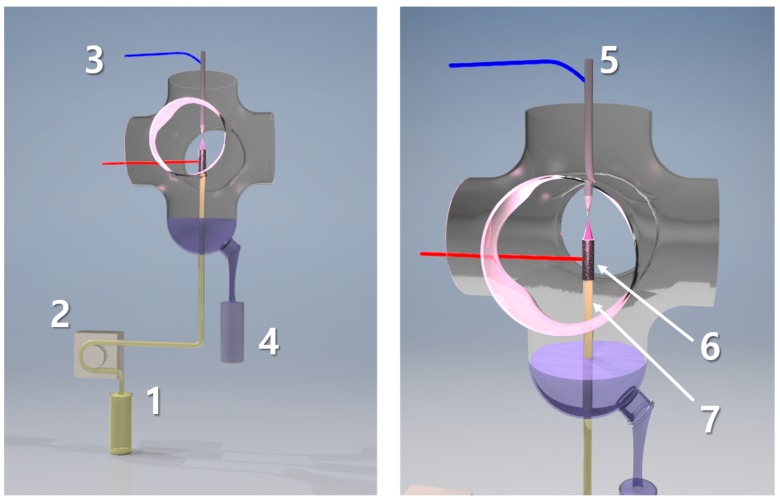
A continuous-flow reaction-discharge system for synthesis of poly(vinylpyrrolidone)-stabilized Pt nanoparticles (PVP-PtNPs); (**1**) the mixed working solution containing the PtNPs precursor and the PVP capping agent (FLA), (**2**) a four-channel peristaltic pump, (**3**) high-voltage wires, (**4**) a collector for the direct current atmospheric pressure glow discharge (dc-APGD)-treated solution, (**5**) a tungsten cathode, (**6**) graphite, and (**7**) quartz tubes.

The mixed working solution of the PtNPs precursor (as Pt(IV) ions at 50 mg L^−1^) and PVP (at 0.25%) was treated by dc-APGD operated in the continuous-flow reaction-discharge system previously reported by Dzimitrowicz et al. [21]. To find the optimal concentration of PVP, the effect of three concentrations of this polymer were examined, i.e., 0.10, 0.25, and 0.50% (*m*/*v*). It was observed that in the presence of PVP at 0.10% (*m*/*v*), visible sedimentation of PtNPs occurred. On the other hand, at 0.50% (*m*/*v*) of PVP, even aggregates occurred. At 0.25% (*m*/*v*) of PVP, no sedimentation, aggregation, nor coalescence of produced PtNPs was observed. Stable dc-APGD was sustained between the surface of the FLA solution and the sharpened pin-type tungsten cathode (Figure 1) in a 90 mm (height) by 40 mm (radial) quartz chamber. The gap between both electrodes was ~5.0 mm. A dc-HV supply (Dora Electronics Equipment, Wroclaw, Poland) was used to provide a HV-positive potential (1100–1300 V) to liquid electrode. The discharge current was maintained at a constant value of 55 mA by applying a ballast resistor with resistance of 10 kΩ (Tyco Electronics, Berwyn, IL, USA). The mixed working solution was introduced to the reaction-discharge system through a quartz-graphite tube at a flow rate of 3.0 mL min^−1^ by using a four-channel peristaltic pump (Masterflex L/S, Cole-Parmer, Vernon Hills, IL, USA), and dc-APGD-treated solutions overflowing the quartz-graphite tube were collected for subsequent analyses. 

Detailed characteristics of the plasma reaction-discharge system with APP generated in contact with a FLA are given elsewhere [34]. The rotational temperature of N_2_ molecules, determined in the liquid-discharge interfacial zone, was considered an approximation of kinetic gas temperature and was about 1400 K, while the vibrational temperature of N_2_ molecules (~5300 K) and the excitation temperature of H atoms (~5200 K) were considerably higher. Differences between temperatures indicated that the developed APGD-based reaction-discharge system was in a high non-equilibrium state. In the spectral range of 200–800 nm, NO, N_2_, N_2_^+^, and OH species were easily excited. Additionally, H and O atomic lines were identified.

### 2.3. Characterization of PVP-PtNPs

Optical properties of PVP-PtNPs present in dc-APGD treated solutions were determined using a Specord 210 (Analytic Jena AG, Jena, Germany) spectrophotometer. The UV/Vis spectra were acquired in the spectral range from 350 to 700 nm at a scanning speed of 20 nm s^−1^ and a step of 0.2 nm. These spectra were recorded 24 h after dc-APGD treatment of mixed working solutions. 

Granulometric properties (size, shape, and elemental composition) of synthesized PVP-PtNPs were assessed using a Tecnai G^2^20 X-TWIN TEM instrument (FEI, Hillsboro, OR, USA), equipped with an EDX microanalyzer (FEI, Hillsboro, OR, USA). TEM and EDX measurements were carried out as follows: one drop of dc-APGD-treated solution was placed onto a Cu grid (CF400-Cu-UL, Electron Microscopy Sciences, Hatfield, PA, USA) and left to dry on air. The average size of PVP-PtNPs was calculated on the basis of the diameters of 100 single nanostructures using FEI Software (version 3.2 SP6 build 421, FEI, Hillsboro, OR, USA).

### 2.4. Surface Functionalization of PtNPs by PVP

To confirm surface functionalization of PtNPs by PVP, plasma-synthesized Pt nanostructures included in collected solutions were characterized using DLS and ATR FT-IR. DLS measurements were performed applying a Zetasizer Nano-ZS instrument (Malvern Instrument, Malvern, UK) with an optical arrangement of the detector at 173° (backscatter angle) and a HeNe laser (633 nm). DLS analyses were carried out in optically homogenous polystyrene cuvettes at temperature of 25 °C. Results (size by number) were evaluated using the ZetaSizer Software (Malvern Dispersion Technology Software, version 7.11) and averaged for three independent runs. ATR FT-IR spectra were acquired in the range from 4000 to 400 cm^−1^, with resolution of 4 cm^−1^ and 64 scans by using a Vertex 70v FTIR spectrophotometer (Bruker, Bremen, Germany). The instrument was equipped with a diamond ATR accessory. 

### 2.5. Application of PVP-PtNPs in the HMS

**Figure 2:**
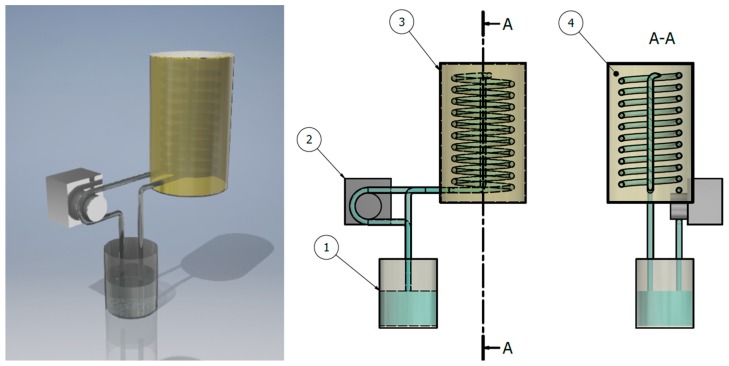
A 3D model of the heat management system (HMS) composed of (**1**) a cooling liquid reservoir, (**2**) a peristaltic pump, (**3**) a spiral radiator filled with (**4**) a conductive layer (the NF containing PVP-PtNPs or water).

The NF containing PVP-PtNPs at a concentration of 0.0008% (*m*/*m*) was used as a conductive layer within a radiator to cool a liquid circulating between the radiator and a simulated internal combustion block. Figure 2 schematically shows the designed HMS. 

The system, as displayed in Figure 2, was composed of a container with cooling liquid (100 mL), a peristaltic pump, and a radiator (150 mm long, diameter 22.5 mm) containing 60 mL of a heat conductive layer (water or the NF). To simulate cooling of the internal combustion block, the container with the liquid was placed on an IKA MAG HS 7 heating plate (Warsaw, Poland), which played the role of the internal combustion chamber, and transferred heat to the liquid. The heating plate was set at a constant power of 200 W, as preliminary tests indicated that this value was sufficient to heat the system to 80 °C. The heated liquid was circulated within the system by a peristaltic pump at a flow rate of 50 mL min^−1^. To monitor the simulated HMS, the procedure was divided into two parts: (i) assessment of the system to control temperature of the cooling liquid at a constant power of the combustion chamber, and (ii) emergency cooling thereof. In the case of heat management, a container filled with water was placed on a heating plate and heated to 80 °C, which was defined as a theoretical border after which a further increase in temperature could cause overheating of the system. Therefore, when the system reached 80 °C, the peristaltic pump was turned on to simulate the HMS. This caused water to be circulated through the system, and resulted in cooling down of the heated water. When it was heated back up to 80 °C, the second part of the procedure was initiated, i.e., emergency cooling. In this case, after the system returned to 80 °C, the heating plate was immediately turned off, and a fan attached to the radiator was turned on to simulate emergency cooling of the internal combustion block. Water was circulated until it was cooled down to 30 °C. The procedure was carried out at ambient temperature (25 °C). Temperatures of water and the heat conductive layer (either the NF or water) were constantly monitored. Recorded temperatures and duration of heating/cooling were used as variables in a simplified version of Newton’s relation between temperatures of the heated/cooled liquid and the surrounding environment [35], defined as dT(t)/dt = k(h/c)∙∆T(t)—where T(t) is temperature at a given time; k(h/c) is the rate of temperature changes, i.e., heating or cooling (s^−1^); and ∆T(t) is a difference in temperature over time t. 

## 3. Results and Discussion

### 3.1. Application of dc-APGD for Synthesis of PVP-PtNPs

**Figure 3:**
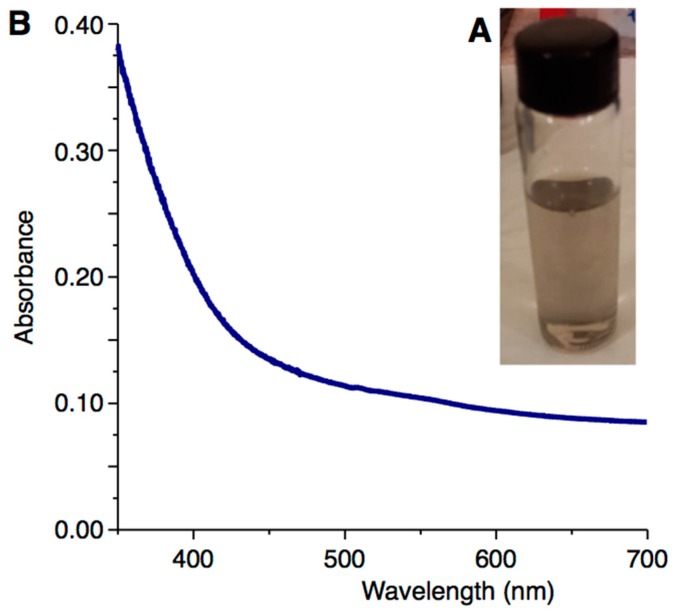
dc-APGD-mediated synthesis of PVP-PtNPs. (**A**) Color exhibited by the mixed working solution after dc-APGD treatment and related to production of PVP-PtNPs; (**B**) the UV/Vis spectrum of PVP-PtNPs.

The first evidence that dc-APGD operated between the surface of the FLA solution and the pin-type tungsten cathode effectively led to continuous-flow synthesis of PVP-PtNPs was the change in color of the mixed working solution treated by the discharge in the studied reaction-discharge system. In these conditions, the solution was observed to change from colorless to black (Figure 3A). According to Wang et al., it was likely associated with formation of PtNPs nuclei in this solution [36]. Moreover, neither aggregation, agglomeration, nor sedimentation of resultant Pt nanostructures was observed (Figure 3A). This first visual observation confirmed successful production of PtNPs stabilized by PVP in the continuous-flow reaction-discharge system used.

### 3.2. Characterization of PVP-PtNPs 

Figure 3B displays the UV/Vis absorption spectrum of the mixed working solution treated by dc-APGD. As can be seen, it presents typical features for Pt nanostructures, i.e., the absorption band occurred across the entire UV/Vis region. Furthermore, absorption increased as the wavelength decreased. As was suggested by Yang et al., this was consistent with the optical properties of PtNPs, and hence, supported the presence of Pt nanostructures synthesized due to plasma-liquid interactions (PLIs) [37].

**Figure 4:**
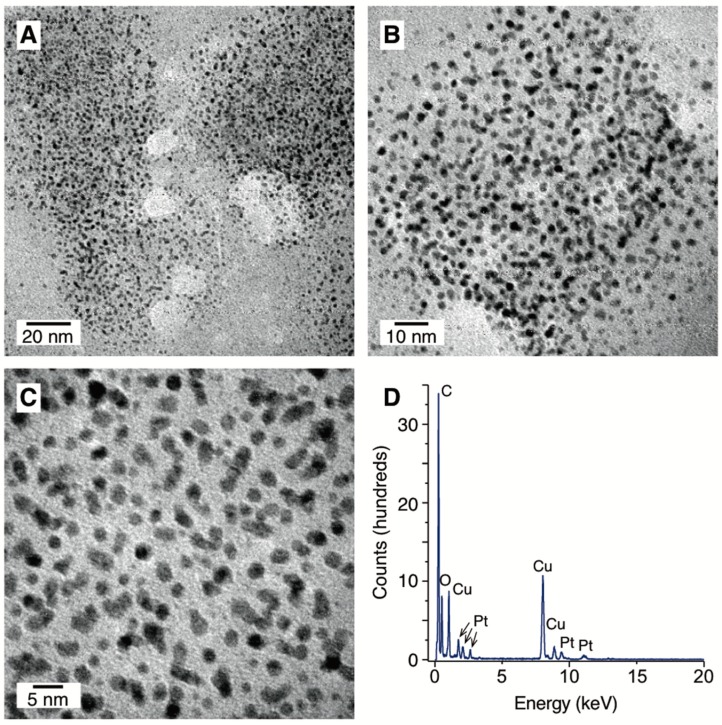
Granulometric properties of PVP-PtNPs synthesized with the aid of dc-APGD. (**A**–**C**) Representative TEM photomicrographs; (**D**) the EDX spectrum.

The morphology and element composition of PVP-PtNPs was determined using TEM and EDX, respectively. On the basis of TEM measurements, the average size of PtNPs was 2.92 nm with a relatively narrow size distribution, i.e., 0.6 nm as standard deviation. TEM images also indicated that the PVP-PtNPs formed were monodisperse, and approximately spherical in shape (Figure 4A–C). Based on the EDX spectrum, the presence of Pt, C, O, and Cu was identified (Figure 4D). Occurrence of metallic Pt resulted from reduction of PtCl_6_^2−^ ions to Pt(0) of nanometric size by dc-APGD-mediated processes in the applied continuous-flow reaction-discharge system. Detection of C and O was likely associated with the chemical structure of PVP. Occurrence of Cu was due to deposition of samples on Cu grids prior to TEM analysis. All these data confirmed that it was possible to produce small (average size of approximately 2 nm) PtNPs through PLIs in the studied reaction-discharge system. This was coincident with results reported by others, who produced PtNPs of ~2 nm in size using different types of APPs [15,17,20]. Nevertheless, the unquestionable advantage of the plasma-based method developed here over other APP-based methods reported in the literature was its continuous-flow character, resulting in high production efficiency of PVP-PtNPs. Accordingly, it was possible to produce 180 mL of PVP-PtNPs per hour in the proposed continuous-flow reaction-discharge system.

**Figure 5:**
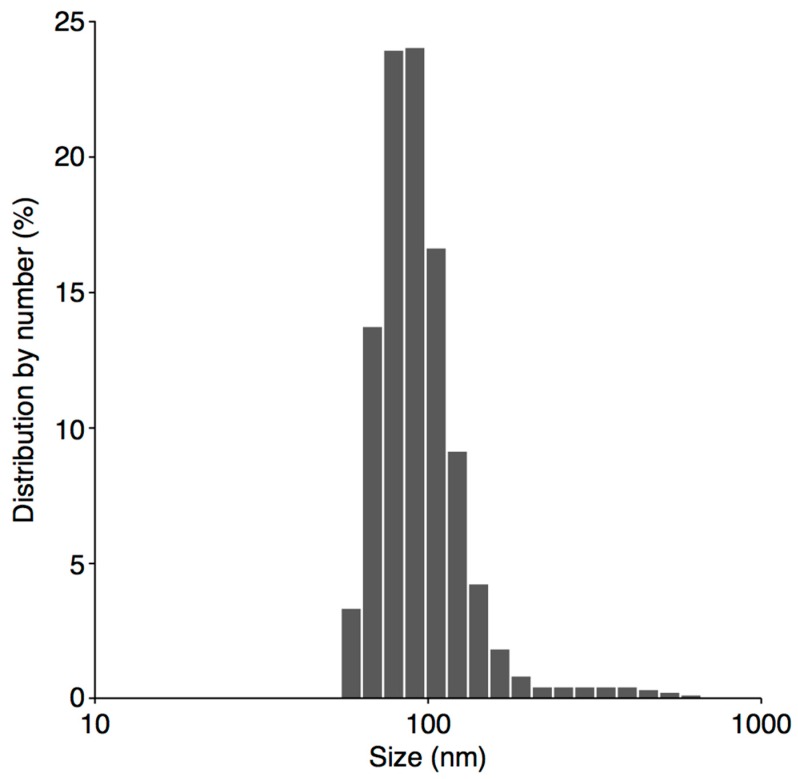
A histogram displaying size by number distribution of PVP-PtNPs as determined by DLS.

**Figure 6:**
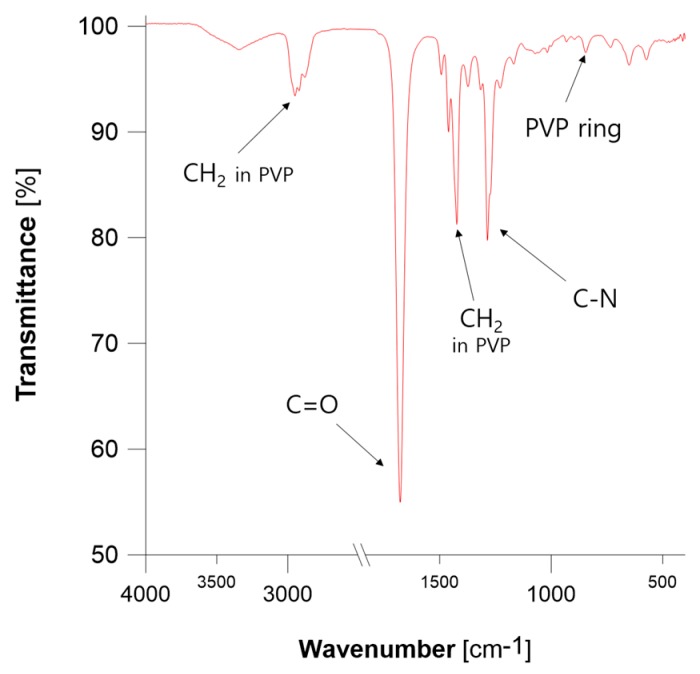
The ATR FT-IR spectrum of PVP-PtNPs.

Surface functionalization of PtNPs with PVP was examined using DLS and ATR FT-IR. Size measurements of resultant Pt nanostructures, as determined by DLS, were much larger than those calculated on the basis of TEM micrographs; the average size by number was 111.4 ± 22.6 nm (Figure 5). This discrepancy in the size of PVP-PtNPs determined using both mentioned techniques was consistent with successful functionalization of Pt nanostructures with PVP [38]. This was because DLS enabled measurement of the hydrodynamic diameter of entire structures, i.e., PtNPs plus attached compounds, whereas TEM measurements were solely based on metallic cores of Pt nanostructures. ATR FT-IR further supported surface functionalization of PtNPs (Figure 6). A sharp, intensive absorption band at 1677 cm^−1^ was observed in the spectrum, and was attributed to C=O stretching vibrations *ν* from the carbonyl group. Absorption bands at 2950, 1423, and 1286 cm^−1^ were assigned to CH_2_ asymmetric stretching vibrations of the aliphatic methylene group and C–N stretching vibrations *ν* of the PVP ring, respectively [39,40,41]. Furthermore, occurrence of the absorption band at 845 cm^−1^ was previously recognized as indicative of the PVP ring oriented towards PtNPs [36]. Moreover, all absorption bands observed in the ATR FT-IR spectrum were shifted by about 20 cm^−1^ in relation to standard charts used for evaluation of characteristic groups and moieties [41]. This effect was already observed [39] and explained by coordination of metallic species by PVP [42]. All these observations confirmed the presence of PVP in Pt NFs, and the role of this capping agent in steric stabilization and functionalization of plasma-synthesized PtNPs. 

TEM micrographs further suggested that the PVP matrix could encapsulate PtNPs. In this case, PtNPs seemed to be purposely dispersed, that is, grouped in approximately spherical regions of over 60 nm in diameter. Formation of PVP capsules containing PtNPs, as opposed to formation of individual PtNPs coated with PVP, could also partly explain differences in the size as measured by TEM and DLS. DLS could measure the size of entire capsules, whereas TEM would reveal the size of individual PtNPs within these capsules. Formation of capsules might be related to physicochemical properties of PVP. This polymer contains N and O atoms bearing free electron pairs that could have ability to chelate surface-active compounds such PtNPs [39,43].

### 3.3. Mechanism of PtNPs Formation

The use of dc-APGD generated in contact with liquid for synthesis of PtNPs is extremely rarely reported in literature. Only Koo et al. and Shim et al. reported to use ac-APGDs operated with the aid of H_2_/He jets in contact with bulky solution reservoirs containing PtCl_6_^2−^ ions for plasma-mediated synthesis of PtNPs [15,16]. These authors hypothesized that PtCl_6_^2−^ ions were possibly reduced by H atoms formed in the solution. This could only be partly correct because much more reactive species, capable of reducing the PtNPs precursor, would be formed as a result of PLIs [44]. In the present work, dc-APGD, fully sustained in surrounding air atmosphere (with no additional gas) and operated in contact with the FLA solution, was used for continuous-flow reduction of PtCl_6_^2−^ ions and synthesis of PtNPs through PLIs. Detailed characteristics of this reaction-discharge system have been previous published, and the produced reactive species characterized [34], allowing us to provide a putative mechanism for the formation of PtNPs. In case of the reaction-discharge system proposed in the present work, there was no voltage fall at the liquid surface and hence, a large flux of electrons from the discharge column bombarded the surface of the FLA solution, leading to production of a very high concentration of interfacial solvated electrons (e_aq_^−^) [45]. These electrons are both highly reactive, and have an anomalously high diffusion constant [45]. Therefore, they could take part in direct reduction of PtCl_6_^2−^ ions in the FLA solution (e.g., PtCl_6_^2−^ + 4e_aq_^−^ = Pt^0^ + 6Cl^−^), resulting in formation of a large number of Pt^0^ nuclei in a very short period of time. Such large number of Pt seeds reached in the FLA solution certainly helped in obtaining smaller in size NPs, as indicated by their morphology as assessed by TEM. Other reactive species could also be formed due to the decomposition of water molecules (in the interfacial zone as well as in the liquid phase, i.e., the FLA solution), including H atoms, OH radicals, or H_2_O_2_ molecules. However, yields of reactions leading to formation of these species should be much lower than observed for the liquid cathode, as reported for dc-APGDs operated using gaseous jets in contact with ionic liquids (ILs) [46].

The role of PVP in the stabilization of the small-sized PtNPs synthesized could be related to their adhesion to nanoparticles through charge-transfer interactions between pyrrolidone rings and the surface of Pt atoms, and formation of >C=O–Pt coordination bonds [47,48]. In this way, PVP-capped PtNPs could be stabilized in two different ways, that is, a polymeric shell could be formed, leading to a structure that prevents further growth and/or agglomeration [47]; in addition, charge transfer could occur [48], making PtNPs negatively charged, and hence, repulsing them in the solution.

### 3.4. Enhanced HMS

**Figure 7:**
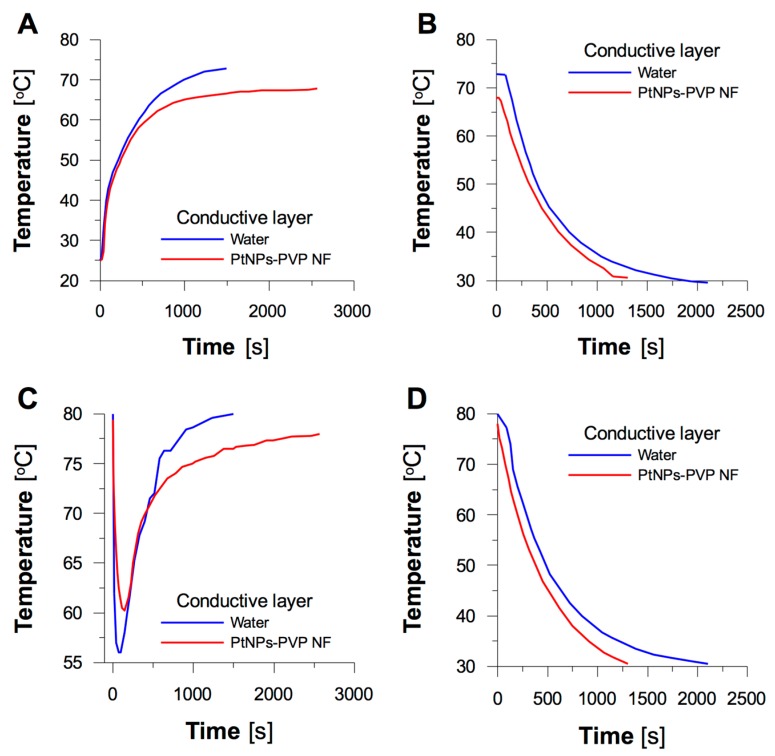
Time-dependent changes of temperature obtained for (**A**) heating, and (**B**) cooling of the conductive layer; (**C**) heating, and (**D**) cooling of the circulating liquid.

To the best of our knowledge, no research on the evaluation of the suitability of NFs containing noble metal NPs for cooling internal combustion chambers have been reported so far. For that reason, a HMS composed of a cooling liquid (water) circulating between a reservoir and a spiral radiator within a layer of the NF containing PVP-PtNPs synthesized by dc-APGD (see Figure 2 for more details) was proposed, and studied in detail. Design of the HMS used in the present work corresponded to common designs of popular liquid-cooling systems applied for managing temperature of combustion blocks [29]. The HMS examined here particularly simulated two scenarios, i.e., (i) suppression of an increase in temperature of the cooling liquid, and (ii) emergency cooling of the internal combustion chamber (see Section 2.4 for more details). The temperature of heating/cooling (T) was measured as a function of time (t), and plotted as shown in Figure 7, for both the cooling liquid (water) and the heat conductive layer (water or the NF with PVP-PtNPs).

When the cooling liquid reached a temperature of 80 °C, the medium of the conductive layer began to circulate within the system, resulting in a decrease in temperature of the cooling liquid. Circulation was continued until the cooling liquid was heated back up to 80 °C. As a result, temperature of the medium of the conductive layer in the radiator also increased; this is shown in Figure 7A,C. As can be seen in Figure 7C, initial temperature (80 °C) within the water reservoir rapidly decreased at the beginning of circulation, because the medium of the conductive layer initially had room temperature. The same phenomenon usually takes place in internal combustion engines, however, valves placed between internal and external circuits of the circulation system allow for proper control of the extent of cooling. Afterwards, the temperature of the cooling liquid was increased, as it is done by internal combustion chambers. As can be seen in Figure 7C, if the medium of the conductive layer in the radiator was water, the cooling liquid in the reservoir was heated back to 80 °C within 25 min. By contrast, when the medium of the conductive layer was the NF containing synthesized PVP-PtNPs, the system was unable to reach 80 °C (provided by a heating plate at 200 W), and instead of this, it reached equilibrium at 78 °C within 43 min. Similar differences were also observed for temperature of the medium of the conductive layer. When the temperature of the circulating cooling liquid increased, the temperature of water in the radiator leveled at 75 °C while temperature of the NF of PVP-PtNPs reached just 67 °C.

When the artificial overheating border was reached, the procedure of emergency cooling was engaged (see Section 2.5 for more details). Although the system with the NF containing PVP-PtNPs did not reach this temperature, the procedure of cooling was initiated at its equilibrium temperature of 78 °C. As can be seen in Figure 7D, the cooling liquid circulating in the system was cooled down to 30 °C within 21 min when the NF containing PVP-PtNPs was applied. This was 14 min faster than when water was used as the medium of the conductive layer. The same tendency was observed when monitoring temperature of water and the NF placed within the radiator (see Figure 7B). All these observations indicated that application of the NF as the conductive layer in the spiral radiator prevented overheating of the system in a fast and effective way. Certainly, the NF-containing PVP-PtNPs displayed an increased heat conductivity within the radiator, as compared to water. To evaluate the efficiency of the system, rate constants of heating (k_h_) and cooling (k_c_) of the liquid circulating within the system were assessed (see Table 1).

When the NF containing PVP-PtNPs was used as the conductive layer, the rate constant responsible for the increase of temperature (k_h_) was almost half the value of this assessed for water, i.e., 2.70 × 10^3^ s^−1^ versus 4.88 × 10^3^ s^−1^. This meant that the presence of the NF significantly extended operation of the system against overheating, as compared to water placed in the radiator. On the other hand, the rate constant of cooling (k_c_) was greater when the NF of PVP-PtNPs was used instead of water, which was responsible for the reduced time needed to cool down the whole system. These differences were certainly reflected by ability of the system to operate within safe temperature. The circulating liquid cooled by water placed in the radiator achieved overheating temperature within 25 min, while the NF containing PVP-PtNPs conveniently prevented overheating of the system.

Based on all observations, it appeared that application of PVP-PtNPs would efficiently facilitate both temperature control of the cooling liquid circulating in the internal combustion block, and emergency cooling thereof. It was previously reported that application of a NF containing 1.5% of Cu and Al_2_O_3_ NPs led to increased heat exchange up to 80%, as compared to water [29,32]. As found in the present work, utilization of the NF containing 0.0008% of PVP-PtNPs resulted in increasing efficiency of the system by 80% during heating, and 30% during cooling, as compared to water. However, it must be remembered that the medium containing PVP-PtNPs was used only as the heat-conductive layer located in the radiator, not as the cooling liquid itself. This significantly reduced the required amount of the NF; hence, the expense for such a system might meet economic requirements. The applied approach overcame two barriers of such systems. Firstly, (1) application of PtNPs led to a significant reduction in the concentration of NPs in the NF needed to successfully manage heat, as compared to already reported systems. Secondly, (2) stabilization of PtNPs by PVP allowed for sealing and immobilization of NF in the radiator; this led to a reduction of the volume of the NF, addressing a potential issue with emission of NPs into the environment.

## 4. Conclusions

It was established that the action of dc-APGD, completely operated in surrounding air only, onto the continuously flowing solution of PtCl_6_^2−^ ions with admixed PVP, led to on-line formation of monodisperse and nearly spherical PVP-capped PtNPs in the liquid phase, with the average diameter of 2.9 ± 0.6 nm. Since the solution of the PtNPs precursor was positively charged and acted as the FLA, it was supposed that solvated electrons were the most important species responsible for reduction of PtCl_6_^2−^. Functionalization of the surface of reduced Pt by PVP resulted in high stability of continuously synthesized and uniformly sized PtNPs. It was also found that the NF containing just 0.0008% of PVP-capped PtNPs could serve as a very promising heat conductive medium for a spiral radiator that efficiently manages heat generated by a simulated combustion chamber. As compared to water, the NF containing PVP-PtNPs resulted in increasing efficiency of such system by 80% and 30% during heating and cooling, respectively.

## Figures and Tables

**Table 1:** Rate constants of heating (k_h_) and cooling (k_c_) of the circulating liquid.

Medium of the Conductive Layer	Rate Constants [×10^−3^ s^−1^]	
	k_h_	k_c_
Water	4.88	2.55
PVP-PtNPs	2.70	3.41

## References

[1] d’Agostino R., Favia P., Oehr C., Wertheimer M.R. (2005). Low-temperature plasma processing of materials: past, present, and future. Plasma Process. Polym..

[2] Weltmann K.D., von Woedtke T. (2017). Plasma medicine—Current state of research and medical application. Plasma Phys. Control. Fusion.

[3] Weltmann K.D., Kindel E., von Woedtke T., Hahnel M., Stieber M., Brandenburg R. (2010). Atmospheric-pressure plasma sources: Prospective tools for plasma medicine. Pure Appl. Chem..

[4] Lu X., Xiong Z., Zhao F., Xian Y., Xiong Q., Gong W., Zou C., Jiang Z., Pan Y. (2009). A simple atmospheric pressure room-temperature air plasma needle device for biomedical applications. Appl. Phys. Lett..

[5] Jamroz P., Greda K., Pohl P. (2012). Development of direct-current, atmospheric-pressure, glow discharges generated in contact with flowing electrolyte solutions for elemental analysis by optical emission spectrometry. TrAC Trends Anal. Chem..

[6] Zhou Z., Huang Y., Yang S., Chen W. (2011). Introduction of a new atmospheric pressure plasma device and application on tomato seeds. J. Agric. Sci..

[7] Oda T. (2003). Non-thermal plasma processing for environmental protection: Decomposition of dilute vocs in air. J. Electrostat..

[8] Cyganowski P., Dzimitrowicz A., Jamroz P., Jermakowicz-Bartkowiak D., Pohl P. (2018). Polymerization-driven immobilization of dc-APGD synthesized gold nanoparticles into a quaternary ammonium-based hydrogel resulting in a polymeric nanocomposite with heat-transfer applications. Polymers.

[9] Dzimitrowicz A., Bielawska-Pohl A., diCenzo G., Jamroz P., Macioszczyk J., Klimczak A., Pohl P. (2018). Pulse-modulated radio-frequency alternating-current-driven atmospheric-pressure glow discharge for continuous-flow synthesis of silver nanoparticles and evaluation of their cytotoxicity toward human melanoma cells. Nanomaterials.

[10] Rioux R., Song H., Grass M., Habas S., Niesz K., Hoefelmeyer J., Yang P., Somorjai G. (2006). Monodisperse platinum nanoparticles of well-defined shape: Synthesis, characterization, catalytic properties and future prospects. Top. Catal..

[11] Choi K.M., Na K., Somorjai G.A., Yaghi O.M. (2015). Chemical environment control and enhanced catalytic performance of platinum nanoparticles embedded in nanocrystalline metal–organic frameworks. J. Am. Chem. Soc..

[12] Cheng N., Banis M.N., Liu J., Riese A., Li X., Li R., Ye S., Knights S., Sun X. (2015). Extremely stable platinum nanoparticles encapsulatedin a zirconia nanocage by area-selective atomic layer deposition for the oxygen reduction reaction. Adv. Mater..

[13] Yadav O., Palmqvist A., Cruise N., Holmberg K. (2003). Synthesis of platinum nanoparticles in microemulsions and their catalytic activity for the oxidation of carbon monoxide. Colloids Surf. A Physicochem. Eng. Asp..

[14] Nagao H., Ichiji M., Hirasawa I. (2017). Synthesis of platinum nanoparticles by reductive crystallization using polyethyleneimine. Chem. Eng. Technol..

[15] Koo I.G., Lee M.S., Shim J.H., Ahn J.H., Lee W.M. (2005). Platinum nanoparticles prepared by a plasma-chemical reduction method. J. Mater. Chem..

[16] Shim J., Joung K.J., Ahn J.H., Lee W.M. (2007). Carbon-supported platinum nanoparticles synthesized by plasma-chemical reduction method for fuel cell applications. J. Electrochem. Soc..

[17] Hu X., Takai O., Saito N. (2013). Simple synthesis of platinum nanoparticles by plasma sputtering in water. Jpn. J. Appl. Phys..

[18] Sato S., Mori K., Ariyada O., Atsushi H., Yonezawa T. (2011). Synthesis of nanoparticles of silver and platinum by microwave-induced plasma in liquid. Surf. Coat. Technol..

[19] Ichin Y., Mitamura K., Saito N., Takai O. (2009). Characterization of platinum catalyst supported on carbon nanoballs prepared by solution plasma processing. J. Vac. Sci. Technol. A..

[20] Dao V.-D., Tran C.Q., Ko S.-H., Choi H.-S. (2013). Dry plasma reduction to synthesize supported platinum nanoparticles for flexible dye-sensitized solar cells. J. Mater. Chem. A.

[21] Dzimitrowicz A., Greda K., Lesniewicz T., Jamroz P., Nyk M., Pohl P. (2016). Size-controlled synthesis of gold nanoparticles by a novel atmospheric pressure glow discharge system with a metallic pin electrode and a flowing liquid electrode. RSC Adv..

[22] Dzimitrowicz A., Jamroz P., Pogoda D., Nyk M., Pohl P. (2017). Direct current atmospheric pressure glow discharge generated between a pin-type solid cathode and a flowing liquid anode as a new tool for silver nanoparticles production. Plasma Process. Polym..

[23] Dzimitrowicz A., Motyka A., Jamroz P., Lojkowska E., Babinska W., Terefinko D., Pohl P., Sledz W. (2018). Application of silver nanostructures synthesized by cold atmospheric pressure plasma for inactivation of bacterial phytopathogens from the genera Dickeya and Pectobacterium. Materials.

[24] Polte J. (2015). Fundamental growth principles of colloidal metal nanoparticles–a new perspective. CrystEngComm.

[25] Das S.K., Choi S.U.S., Patel H.E. (2006). Heat transfer in nanofluids—A review. Heat Transfer Eng..

[26] Patel H.E., Das S.K., Sundararajan T., Sreekumaran Nair A., George B., Pradeep T. (2003). Thermal conductivities of naked and monolayer protected metal nanoparticle based nanofluids: Manifestation of anomalous enhancement and chemical effects. Appl. Phys. Lett..

[27] Wang P., Lv J., Bai M., Li G., Zeng K. (2015). The reciprocating motion characteristics of nanofluid inside the piston cooling gallery. Powder Technol..

[28] Kajiwara H., Fujioka Y., Negishi H. (2003). Prediction of temperatures on pistons with cooling gallery in diesel engines using CFD tool. SAE Tech. Pap..

[29] Hatami M., Ganji D., Gorji-Bandpy M. (2014). A review of different heat exchangers designs for increasing the diesel exhaust waste heat recovery. Renew. Sustain. Energy Rev..

[30] Xuan Y., Li Q. (2000). Heat transfer enhancement of nanofluids. Int. J. Heat Fluid Flow.

[31] Kakac S., Pramuanjaroenkij A. (2009). Review of convective heat transfer enhancement with nanofluids. Int. J. Heat Mass Transf..

[32] Moghaieb H.S., Abdel-Hamid H.M., Shedid M.H., Helali A.B. (2017). Engine cooling using Al_2_O_3_/water nanofluids. Appl. Therm. Eng..

[33] Tsai C.Y., Chien H.T., Ding P.P., Chan B., Luh T.Y., Chen P.H. (2004). Effect of structural character of gold nanoparticles in nanofluid on heat pipe thermal performance. Mater. Lett..

[34] Greda K., Swiderski K., Jamroz P., Pohl P. (2016). Flowing liquid anode atmospheric pressure glow discharge as an excitation source for optical emission spectrometry with the improved detectability of Ag, Cd, Hg, Pb, Tl, and Zn. Anal. Chem..

[35] Burmeister L.C. (1993). Solutions manual. Convective Heat Transfer.

[36] Wang C., Daimon H., Onodera T., Koda T., Sun S. (2008). A general approach to the size-and shape-controlled synthesis of platinum nanoparticles and their catalytic reduction of oxygen. Angew. Chem. Int. Ed..

[37] Yang W., Ma Y., Tang J., Yang X. (2007). “Green synthesis” of monodisperse Pt nanoparticles and their catalytic properties. Colloids Surf. A Physicochem. Eng. Asp..

[38] Chytil S., Glomm W.R., Vollebekk E., Bergem H., Walmsley J., Sjoblom J., Blekkan E.A. (2005). Platinum nanoparticles encapsulated in mesoporous silica: Preparation, characterisation and catalytic activity in toluene hydrogenation. Microporous Mesoporous Mater..

[39] Abdelghany A., Mekhail M.S., Abdelrazek E., Aboud M. (2015). Combined DFT/FTIR structural studies of monodispersed PVP/Gold and silver nano particles. J. Alloys Compd..

[40] Song Y.-J., Wang M., Zhang X.-Y., Wu J.-Y., Zhang T. (2014). Investigation on the role of the molecular weight of polyvinyl pyrrolidone in the shape control of high-yield silver nanospheres and nanowires. Nanoscale Res. Lett..

[41] Long D.A. (2004). Infrared and Raman characteristic group frequencies. Tables and charts George Socrates John Wiley and sons, ltd, chichester, third edition, 2001. J. Raman Spectrosc..

[42] Kim J.H., Min B.R., Kim C.K., Won J., Kang Y.S. (2002). Spectroscopic interpretation of silver ion complexation with propylene in silver polymer electrolytes. J. Phys. Chem. B.

[43] Cyganowski P., Lesniewicz A., Polowczyk I., Checmanowski J., Kozlecki T., Pohl P., Jermakowicz-Bartkowiak D. (2018). Surface-activated anion exchange resins for synthesis and immobilization of gold and palladium nano- and microstructures. React. Funct. Polym..

[44] Chen C., Li J.S., Li Y.F. (2015). A review of plasma-liquid interactions for nanomaterials synthesis. J. Phys. D Appl. Phys..

[45] Bruggeman P.J., Kushner M.J., Locke B.R., Gardeniers J.G.E., Graham W.G., Graves D.B., Hofman-Caris R.C.H.M, Maric D., Reid J.P. (2016). Plasma-liquid interactions: A review and roadmap. Plasma Sources Sci. Technol..

[46] Hofft O., Endres F. (2011). Plasma electrochemistry in ionic liquids: An alternative route to generate nanoparticles. Phys. Chem. Chem. Phys..

[47] Borodko Y., Habas S.E., Koebel M., Yang P., Frei H., Somorjai G.A. (2006). Probing the interaction of poly(vinylpyrrolidone) with platinum nanocrystals by UV-Raman and FTIR. J. Phys. Chem. B.

[48] Qiu L., Liu F., Zhao L.Z., Yang W.S., Yao J.N. (2006). Evidence of a unique electron donor-acceptor property for platinum nanoparticles as studied by XPS. Langmuir.

